# A quantitative interspecies comparison of the respiratory mucociliary clearance mechanism

**DOI:** 10.1007/s00249-021-01584-8

**Published:** 2022-01-24

**Authors:** Andreas Burn, Martin Schneiter, Manuel Ryser, Peter Gehr, Jaroslav Rička, Martin Frenz

**Affiliations:** 1 Institute of Applied Physics, University of Bern, Sidlerstrasse 5, 3012 Bern, Switzerland; 2 Institute of Anatomy, University of Bern, Baltzerstrasse 2, 3012 Bern, Switzerland

**Keywords:** Cilia, Mucociliary clearance, Metachronal wave, Ciliary beating frequency, Metachronism, High-speed video microscopy, Avian respiratory system

## Abstract

Collectively coordinated ciliary activity propels the airway mucus, which lines the luminal surface of the vertebrate respiratory system, in cranial direction. Our contemporary understanding on how the quantitative characteristics of the metachronal wave field determines the resulting mucociliary transport is still limited, partly due to the sparse availability of quantitative observational data. We employed high-speed video reflection microscopy to image and quantitatively characterize the metachronal wave field as well as the mucociliary transport in excised bovine, porcine, ovine, lapine, turkey and ostrich samples. Image processing techniques were used to determine the ciliary beating frequency (CBF), the velocity and wavelength of the metachronal wave and the mucociliary transport velocity. The transport direction was found to strongly correlate with the mean wave propagation direction in all six species. The CBF yielded similar values (10–15 Hz) for all six species. Birds were found to exhibit higher transport speeds (130–260 $\upmu$m/s) than mammals (20–80 $\upmu$m/s). While the average transport direction significantly deviates from the tracheal long axis in mammals, no significant deviation was found in birds. The metachronal waves were found to propagate at about 4–8 times the speed of mucociliary transport in mammals, whereas in birds they propagate at about the transport speed. The mucociliary transport in birds is fast and roughly follows the TLA, whereas the transport is slower and proceeds along a left-handed spiral in mammals. The longer wavelengths and the lower ratio between the metachronal wave speed and the mucociliary transport speed provide evidence that the mucociliary clearance mechanism operates differently in birds than in mammals.

**Supplementary Information:**

The online version contains supplementary material available at 10.1007/s00249-021-01584-8.

## Introduction

When examining the literature on the respiratory mucociliary clearance system (e.g. see Sleigh et al. 1988; Satir and Sleigh 1990; Priel 1997; Houtmeyers et al. 1999; Wanner et al. 1996; Salathe 2001; Stannard and O’Callaghan 2006; Satir and Christensen 2007; Salathe 2007; Smith et al. 2008; Norton et al. 2011; Elgeti and Gompper 2013; Brumley et al. 2014; Cicuta 2020; Gilpin et al. 2020) one becomes aware of a surprising discrepancy: on one hand, thanks to the recent advances in genomics and proteomics, there is a large body of knowledge on the molecular composition and detailed structure of cilia and mucus. A particularly spectacular example represents the elucidation of the structure and function of the dynein molecular motors, as e.g. reported in Burgess et al. (2003). On the other hand, our understanding of the basic mesoscopic function of the system still appears rather limited: How are the oscillations of a myriad of tightly packed individual cilia translated into a directed movement of the overlying mucus? It is obvious that in order to achieve efficient directed transport the ciliary motions must be at least locally coordinated, forming thereby so-called ‘metachronal waves’ (Knight-Jones 1954). But how this collectively coordinated motion pattern is achieved and how it affects the transport, is not yet fully understood.

One major reason for our limited knowledge is the lack of adequate observations under native conditions in the respiratory tract. Even in excised trachea samples the observation of the ciliary activity is difficult, because of the dense packing of cilia and because the observation is only possible through the corrugated mucus layer above an irregularly formed epithelium topology. Under native conditions the dominant source of contrast is the reflection from the air-mucus interface (see Iravani and Melville 1976 and references therein). The reliability of such indirect observation however became questioned and the research therefore focused on transparent models, which are accessible by transmission microscopy, such as epithelial sheets from frog pharyngal or esophagal epithelium (Wilson et al. 1975; Eshel and Priel 1987; Gheber and Priel 1994), cultured rabbit tracheal epithelium (Cheung and Jahn 1976; Sanderson and Sleigh 1981; Romet et al. 1991) or cultures from human biopsies (Marino and Aiello 1982; Rautiainen et al. 1992; Hard et al. 1999; Chilvers and O’Callaghan 2000). In principle, such models allow high resolution imaging of cilia as well as the analysis of their motion pattern. Typically, Nomarski differential interference contrast is combined with high-speed recordings; in the past with film cinematography (Wilson et al. 1975), today with digital area sensors (Chilvers and O’Callaghan 2000; Sears et al. 2015; Quinn et al. 2015). In practice, however, high resolution recording of densely packed cilia is difficult even with model systems suited for transmission microscopy and therefore, imaging is often supplemented by auxiliary techniques. For example, Eshel and Priel (1987) and Gheber and Priel (1994) used cleverly arranged fiber optic probes to detect the light transmitted through the cell layer, and from the cross-correlation of their signals they determined the wavelength as well as the velocity of the metachronal waves. On the other hand, Sanderson and Sleigh, in their classical work on cultured rabbit tracheal epithelium (Sanderson and Sleigh 1981), combine high-speed cine photography with the ingenious, albeit somewhat indirect technique of fixation of the metachronal wave and subsequent electron-microscopic observation. The picture which emerged from these observations, and which is now found in reviews and textbooks, is the following: mucus propulsion is achieved through the asymmetry of the ciliary motion cycle, which is composed of the so-called ‘recovery stroke’, during which the cilium bends and sweeps clockwise near the cell surface. Subsequent to the ‘recovery stroke’ follows the ‘effective stroke’, during which the cilium moves perpendicularly to the cell surface in an extended configuration, engaging thereby the overlying mucus carpet. The motion of individual cilia are collectively coordinated in antilaeoplectic fashion, that is the metachronal waves move backwards and sidewards with respect to the direction of the effective stroke. Thereby, it is implicitly assumed that the direction of the effective stroke coincides with the direction of the mucus transport. By ‘implicitly assumed’ we would like to advert to a problem: it turns out that most of the evidence was obtained with samples immersed in a medium under a cover slip. Such models lack an essential ingredient, namely the air-exposed mucus layer, which gets propelled by the underlying collectively coordinated ciliary motion. The lack of the air-exposed mucus layer is unfortunate, since it has been plausibly proposed that the coordination of the cilia is the result of hydrodynamic coupling through the viscoelastic working medium (Gueron et al. 1997; Elgeti and Gompper 2013; Brumley et al. 2014). More specifically, it has been shown that an increase of the viscosity of the tissue culture medium (from 20 to 1500 cp) may change the metachronism in cultured frog esophagus from diaplectic to orthoplectic (Gheber et al. 1998).

For this study, we developed a set of techniques and algorithms that allowed us to simultaneously measure the mucociliary transport velocity and the space-time structure of the metachronal wave field on excised, but otherwise unaltered, tracheas under close-to-native environmental conditions. Of course, when observing through the air-mucus interface we do not see the motions of the individual cilia. However, we have shown that if one refrains from using the traditional Nomarski technique, but optimizes the reflective optics instead, then one is able to measure the minute modulation of the air-mucus interface with an adequate sensitivity (Ryser et al. 2007). It is highly likely that these modulations reflect the submucosal ciliary activity, including the metachronal coordination, rather directly, as long as the depth of the airway surface liquid is of the same order of magnitude as the ciliary length. Considering the low Reynolds number of the involved hydrodynamics (Re $\approx$ $10^{-3}$), it is hard to imagine anything else but linear coupling between the surface modulations and the underlying collectively coordinated ciliary activity.

A detailed description of our experimental setup, the image processing algorithms and their validation have previously been presented (Ryser et al. 2007). Here we present the results of a comprehensive comparative study performed with mammalian (bovine, porcine, ovine and lapine) as well as avian (turkey and ostrich) trachea explants.

The original aim of the present study was twofold: (1) to establish a generally valid empirical cross-species model for the interrelationship between the metachronal wave field and the associated mucociliary transport, and (2) to elaborate a comprehensive list of reference values for observables, which quantitatively characterize the metachronal wave field as well as the achieved mucociliary transport.

## Methods

The measurements were performed on an upright bright field microscope equipped with a digital high-speed camera. The microscope was operated in reflection contrast mode using epi-illumination, which allowed us to image modulations of the mucus surface. During the measurements the trachea samples were held in a specially designed climate chamber, which provides standardized environmental conditions over extended time spans of up to several hours.

### Tissue sample preparation

**Fig. 1:**
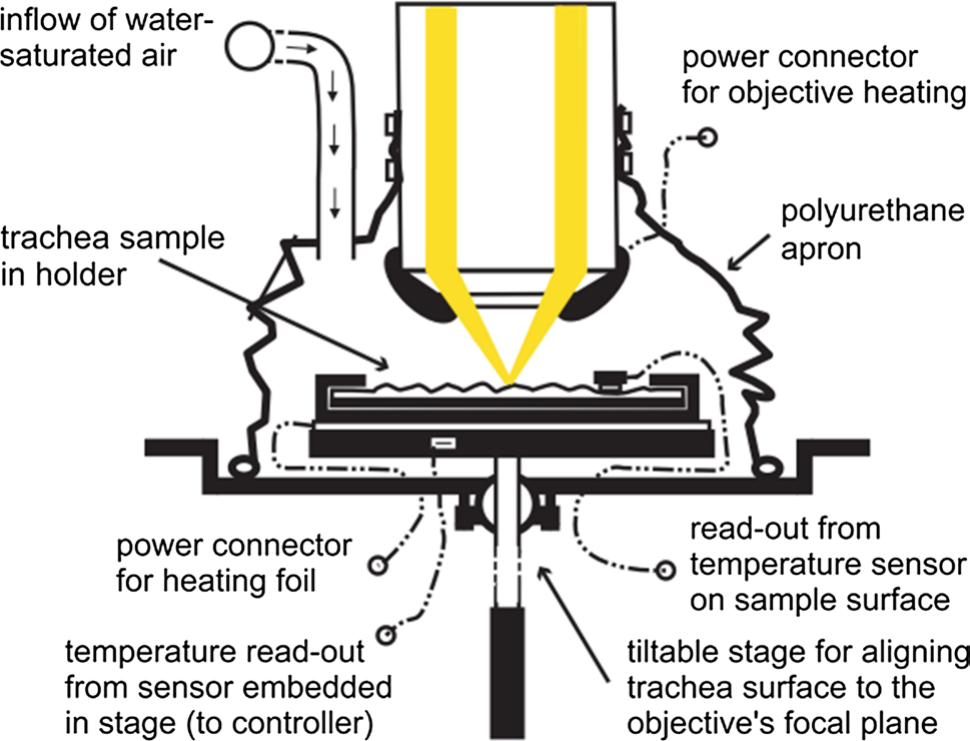
Illustration of the experimental setup. Detailed view of the climate chamber, which is built around a multi-axis adjustable heated microscope stage with removable sample holder and surface temperature sensor and a heated microscope objective. The chamber wall is made of a polyurethane apron. The hollow-beam epi-illumination is indicated in yellow

Fresh tracheas were obtained from the local slaughterhouse, where they were excised about 20 min after the animal’s death and immediately packed into sealed plastic bags. The tracheas were always removed together with the larynx and a part of the upper bronchi, in order to protect the mucosa from being contaminated by blood. Further, it was helpful for keeping track of the gross anatomical orientation of the trachea samples during the preparation process. The tracheas were kept at temperatures below 10 $^\circ$C during transport (15 min) and subsequently stored at 4 $^\circ$C until measurements were performed; approximately 90 min after the animal’s death. Prior to measurement, fat and connective tissue were trimmed away. Tracheal samples measuring 5–25 cm$^2$ in size were cut out of the anterior wall of the central region of the trachea. The apical surface was neither touched nor washed at any time during the preparation process, in order to avoid alterations of the air-liquid interface and the surfactant layer. To flatten the trachea pieces, they were inserted in the slits of a U-shaped sample holder with a brim (see Fig. 1). For all measurements, the trachea samples were cut and fixed on the microscope stage with the cranial end pointing up or away from the observer. This convention on the samples’ orientation was kept during the whole analysis process and was also used in all of the images shown. Shortly before the measurement was started, puffball (calvatia excipuliformis) spores with a diameter of approximately 3.5 $\upmu$m, used as tracer particles for the transport measurement, were blown onto the mucus surface of the trachea sample.

### Imaging setup

An adequate contrast technique is crucial for the observation of the collective mucociliary dynamics. Excised tracheal epithelial tissue is characterized by densely packed and mucus-covered cilia lawns and is very limited in its transparency to visible light. An observation of the ciliary beating by transmission microscopy from the apical side is therefore not practicable. The air–liquid interface at the mucus surface however, opens up the possibility to observe the modulation of the mucus surface by using reflection microscopy. The typical surface modulation measures around 4–5 $\upmu$m vertically, over a planar distance of about 50 $\upmu$m (Ryser et al. 2007). Those dynamic mucus surface modulations were imaged and recorded by using high-speed video reflection microscopy, as previously presented in detail (see Burn 2009; Ryser et al. 2007).

**Fig. 2:**
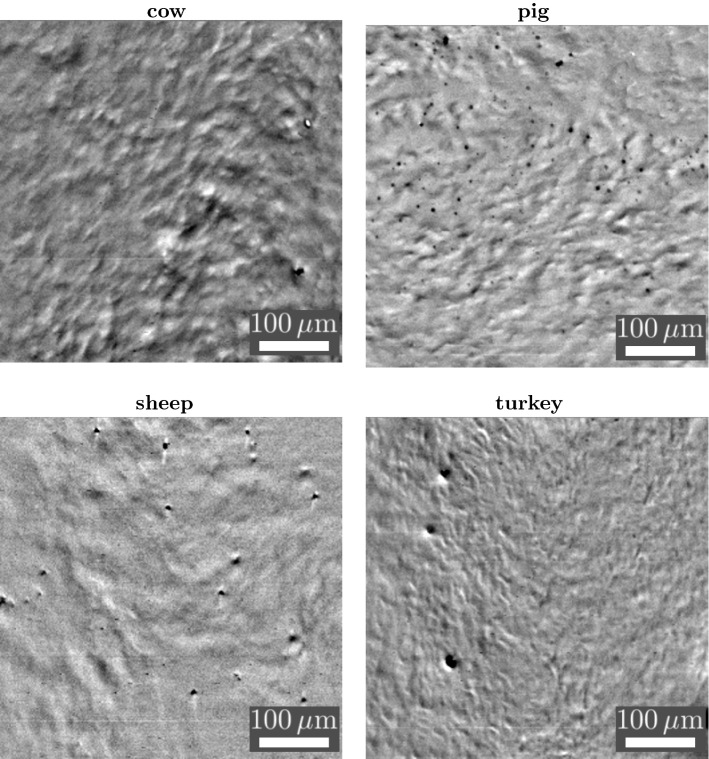
Snapshots of the supplementary enclosed videos taken with our reflectometric microscope setup showing the transport of mucus-embedded tracer particles and the wave-like mucus modulations caused by the underlying metachronally coordinated ciliary activity

Our experimental setup, including the in-house constructed climate chamber, is built around an upright microscope (Nikon Eclipse E600FN, Kanagawa, Japan) with epi-illumination (100 W halogen lamp). A near-infrared filter was inserted into the illumination beam path, in order to protect the tissue sample from heat radiation. Most recordings were taken with the digital CCD camera Dalsa CA-D1T, which offers a high sensitivity, 12-bit digitization and a frame rate of 500 Hz (see Ryser et al. 2007 for further details). Finally, an area of 456 $\times$ 456 $\upmu$m$^2$ was captured by using a 10 $\times$ bright field objective (Nikon Plan Fluor, NA 0.3, WD 16 mm). For illustration, four typical image sequences are provided as supplementary material (see video S1 to video S4, which are dynamically filtered and slightly contrast enhanced). These videos show the wave-like modulations of the mucus surface as well as mucociliary transport, both of which being caused by the underlying metachronally coordinated ciliary activity. In Fig. 2, a snapshot of each supplementary enclosed video is shown.

### Climate control

One of the key requirements for the measurement system was the possibility to maintain the sample under controlled and stable environmental conditions, since temperature and humidity have been shown to influence mucociliary activity (e.g. see Sears et al. 2015; Kilgour et al. 2004; Mercke 1975). The samples were therefore held in a climate-controlled chamber during measurement. The main parts of the system are presented in Fig. 1. Humidity control was achieved by guiding vapor-saturated air from a humidifier into the measurement chamber. Care was taken that the relative humidity was higher than 95% at all times during measurements.

The microscope stage and the sample holder were uniformly heated by a heating foil. A massive aluminum microscope stage and firm fixation of the sample holder guaranteed a homogeneous temperature distribution. Good heat conductance between the temperature controlled microscope stage and the sample was assured by filling possible air gaps between tissue and sample holder with serum free medium (OptiMEM I, GIBCO/Invitrogen, Carlsbad, USA). The temperature of the stage was controlled by a PT100 temperature sensor integrated into the microscope stage and by a temperature controller module. The measurement of the surface temperature of the sample was performed by a second PT100 temperature sensor in contact with the surface of the tissue. Care was taken to maintain a minimum distance of 5 mm between the sensor and the measurement zone in order to prevent disturbance of the mucociliary activity.

The holder with the sample was placed on the pre-heated microscope stage and the sample was allowed to equilibrate with the measurement environment. The temperature was set to 30 $^\circ$C for all species in order to prevent temperature-coupled effects from interfering with the interspecies differences we were interested in. The chosen temperature of 30 ± 0.1 $^\circ$C is slightly lower than the measured in-vivo surface temperature of about 34 $^\circ$C in the upper airways (Ingelstedt 1956; Dery 1973). We preferred the lower value in order to prolong the viability of the samples.

### Sample population

For the present study, data from six different animal species: cow, pig, sheep, rabbit, turkey and ostrich have been acquired. Recordings were taken at different positions within the central region of the trachea. The final pool of data was acquired from 10 to 24 individual tracheas per species with typically 4–7 recordings per trachea (see Table 1). Due to the limited availability, however, only three ostrich tracheas, which delivered consistent data, could be examined. For the statistical analysis, we used only those recordings, for which the complete set of key observables could be determined simultaneously. The set of key observables is represented by: the ciliary beating frequency (CBF), the metachronal wavelength ($\lambda$), the wave propagation velocity ($\vec {v_w}$) and the mucociliary transport velocity ($\vec {v_t}$).

**Table 1:** Overview on the data set: total number of recordings and number of individuals (tracheas) per species used for analysis

	Cow	Pig	Sheep	Rabbit	Turkey	Ostrich
No recordings	56	45	66	100	38	40
No tracheas	14	11	10	24	11	3

## Data analysis

The raw reflectometric image sequences contain three sources of information: (1) the transport information, which is extracted from the trajectories of tracer particles (applied puff ball spores or other debris) transported within the mucus, (2) the collectively coordinated ciliary activity, which is inferred from the dynamic wave-like modulations of the mucus surface and (3) static patterns (like the tracheal topography). The transport information and the metachronal wave field were to be analyzed, whereas the static information was of no particular interest. We developed a dedicated image pre-processing in order to separate the above-mentioned sources of information as well as sophisticated image processing algorithms to ascribe a set of physical observables, or parameters, to each video. This way, a specific set of observable values is meant to characterize the collective mucociliary activity in the associated video. A detailed description of the data processing techniques has previously been published in Ryser et al. (2007). Therefore, we only outline the set of determined observables together with a brief interpretation.

**Fig. 3:**
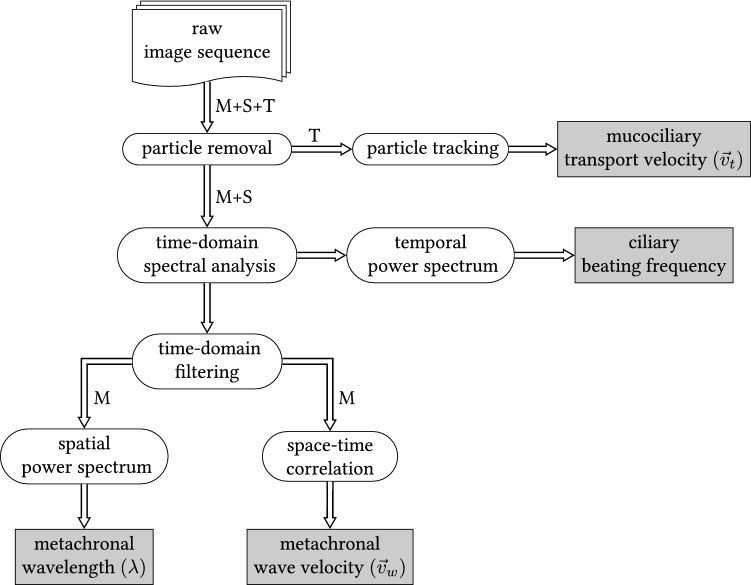
Flowchart of the process for determining the set of observable values conducted for each single recorded image sequence. The capital letters stand for the three sources of information contained in each raw image sequence: T represents the transport information, M the metachronal wave field and S represents static features. The set of determined physical observables consists of the mucociliary transport velocity ($\vec {v}_t$), the ciliary beating frequency, the metachronal wavelength ($\lambda$) and the mean metachronal wave propagation velocity ($\vec {v}_w$). These observables represent the output of the processing algorithms and are therefore shown in rectangular boxes colored in gray

The flowchart shown in Fig. 3 illustrates the procedure of determining the observable values for a single recording. The mucociliary transport velocity ($\vec {v_t}$) represents the mean velocity (speed and direction) of 3–12 tracked tracer particles, which are carried with the mucus. The CBF represents the characteristic ciliary motion frequency and was calculated from the area-averaged temporal power spectrum. The spatial periodicity of the metachronal wave field, i.e. the metachronal wavelength $\lambda$, was inferred from the average spatial power spectrum, and was determined as the inverse of the modus of the distribution of the wave vector magnitudes. Finally, the mean wave propagation velocity ($\vec {v}_{w}$) was determined by tracking the peak cross-correlation in the average space-time correlogram. This observable therefore measures the propagation velocity of the mean dynamic structure and represents the average propagation speed and direction of the waves in the metachronal wave field.

## Results

### Statistical analysis

We carefully analyzed the data for intraspecies differences. It turned out that, for most individuals, the variation in measured parameter values within one trachea is as big as the full variation over all tracheas within the same species. This means that individuals of the same species are not significantly distinguishable from each other. Based on this reasoning, we abandoned the categorial variable individual and weighted all measurements from the same species equally.^1^

For all observables, standard descriptive statistics like the median, the quartiles, and a 95% confidence interval for the median, based on bootstrapped data (Efron and Tibshirani 1986), are indicated in the following. All statistical calculations were made with the ‘R’ software package (R Core Team 2000). In order to not make any assumptions on the distribution of the measured observable values, we used non-parametric tests. For the establishment of an interspecies ranking, the Wilcoxon–Mann–Whitney test (Wilcoxon 1945; Mann and Whitney 1947) was used. In order to compare two independent samples of the same observable, one-tailed hypothesis tests were performed. The hypothesis and its corresponding alternative are as follows:

$$H_0$$:
Population A shows smaller values than population B.
$$H_1$$:
Population A shows greater values than population B.

Differences were regarded as significant, if the probability for a type I error was lower than five percent ($\alpha$ = $5\%$). In the case of the ranking presented, multiple pairwise testing was necessary which results in an accumulation of the chances of committing a type I error (Bonferroni 1935, 1936). Therefore, to take account for the multiple testing, a more restrictive level of significance ($\alpha _{\mathrm{corr}}$ = $0.5\%$) was applied in each individual test. Further, in order to test for interspecies differences, we used the Kruskal–Wallis test (Kruskal and Wallis 1952; Hollander and Wolfe 1973).

### Mucociliary transport velocity $(\vec {v_t})$

For each recording, we ascribed a (local) mucociliary transport velocity, denoted by $\vec {v_t}$, which represents the average velocity of 3–12 tracked tracer particles measured with respect to the TLA. From the vectorial mucociliary transport velocity $\vec {v_t}$, we determined three quantities: (1) the mucociliary transport speed $|\vec {v_t}|$, (2) the mucociliary transport direction and (3) the component of the mucociliary transport velocity pointing along the tracheal axis, denoted by $\langle \vec {v_t}, \hat{\vec {e}}_{\mathrm{TLA}}\rangle$. The latter directly measures the efficacy of the tracheal clearance, and is therefore referred to as the mucociliary clearance speed in the following.

**Fig. 4:**
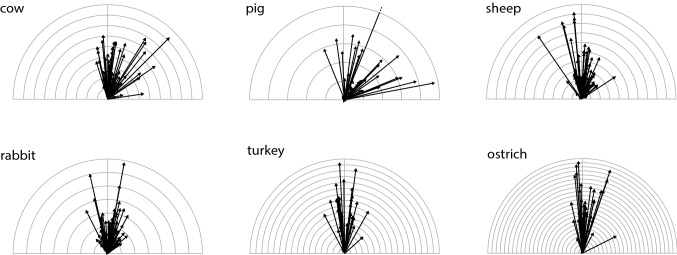
Compilation of the observed mucociliary transport velocities ($\vec {v_t}$) from all measured samples and species. The tick interval corresponds to 20 $\upmu$m/s. Note that the graph is only an overview. The frequency of the occurrence is only marginally indicated, since many arrows are overprinted. This remark applies to all arrow representations displayed in the following

A general overview of the observed mucociliary transport velocities is provided in Fig. 4. A closer look at the data on the mucociliary transport (MCT) direction (see Table 2) shows that in all examined mammalian species, the median MCT direction significantly deviates from the TLA, meaning that the transport runs along a left-handed helical trajectory towards the larynx. In the two examined avian species, however, the MCT direction did not significantly deviate from the TLA.

**Table 2:** Overview of the observed MCT directions (in $^{\circ }$) with respect to the TLA, MCT speeds (in $\upmu$m/s) and MCC speeds (in $\upmu$m/s)

	Cow	Pig	Sheep	Rabbit	Turkey	Ostrich
MCT direction						
Median	9	38	14	9	6	3
95% CI	[5, 17]	[27, 43]	[7, 23]	[5, 16]	[$-$ 6, 9]	[$-$ 1, 6]
MCT speed, $|{\vec{v}}_t|$						
Median	61	27	52	29	153	216
95% CI	[53, 78]	[19, 48]	[49, 66]	[26, 32]	[130, 182]	[189, 260]
MCC speed, ${\langle {{\vec{v}}}_t, {{\hat{\vec{e}}}}_{\mathrm{TLA}} \rangle }$						
Median	53	21	49	25	145	215
95% CI	[42, 70]	[15, 25]	[40, 61]	[22, 29]	[125, 178]	[185, 250]

The median and its 95% CI are listed for each species

**Fig. 5:**
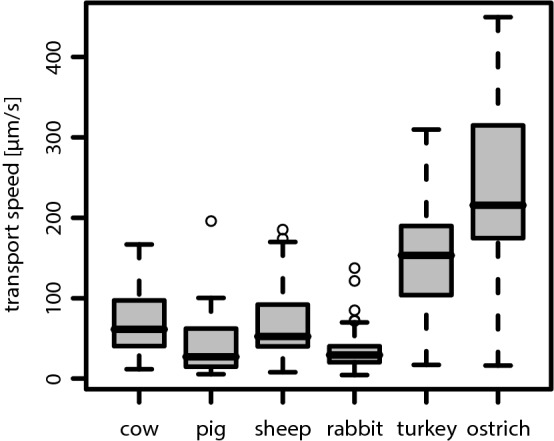
Boxplot of the measured mucociliary transport speed $|\vec {v}_t|$ for each species. Whiskers mark all values lying no further than 1.5 times the interquartile range (IQR), either from below the first, or, from above the third quartile. Outliers are shown by circles. This convention also applies to all of the following boxplots

An overview of the measured values for the mucociliary transport speed $(|\vec {v_t}|)$ is shown in Fig. 5. In comparison to the transport directions, even more pronounced differences were found in terms of the transport speeds (see also Table 2). Among mammals, one recognizes significant differences between two groups: cow and sheep vs. pig and rabbit (Kruskal–Wallis: $p=3.5\cdot 10^{-14}$). As it can clearly be seen in Fig. 5, as well as in Table 2, both avian species show extraordinarily high mucociliary transport speeds being around 3–7 times faster than in mammalian species. As can be seen in the third row of Table 2, which lists the values for the mucociliary clearance speed, the bird-mammal difference is even more pronounced when considering the actual clearance efficacy.

### Ciliary beating frequency (CBF)

**Fig. 6:**
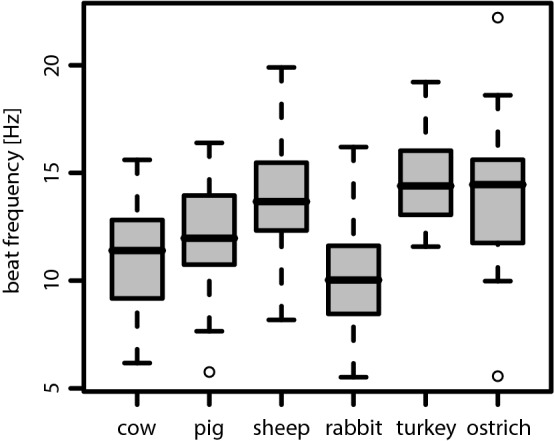
Boxplot of the measured ciliary beat frequencies for each of the six species

All measured CBF values lie within a rather narrow frequency band. The species-specific median CBF values roughly range from 10 to 15 Hz (see Table 3). Even though statistically significant differences between species were found, the interspecies variation is rather small (see also Fig. 6).

**Table 3:** Overview of the measured values for the ciliary beating frequency (CBF) in [Hz]

	Cow	Pig	Sheep	Rabbit	Turkey	Ostrich
CBF [Hz]						
Median	11.4	12.0	13.7	10.0	14.4	14.5
95% CI	[9.9, 12.0]	[11.5, 12.8]	[13.2, 14.9]	[9.2, 10.6]	[13.9, 15.2]	[13.4, 15.2]

The median CBF and its 95% CI are listed for each species

### Mean wavelength of the metachronal wave field $\left( \lambda \right)$

**Fig. 7:**
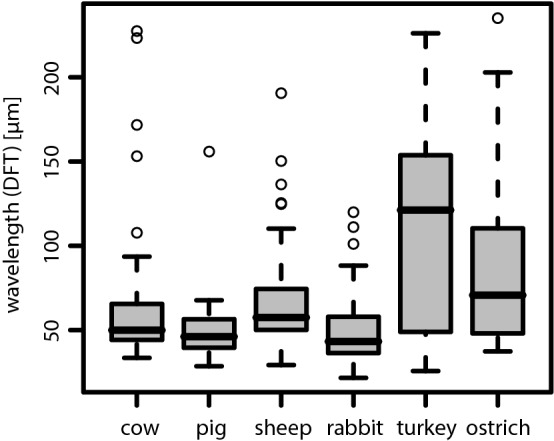
Boxplot of the wavelengths $\lambda$, as determined from the spatial Fourier analysis, for each of the six examined species

As reported previously (Ryser et al. 2007), the spatio-temporal structure of the metachronal wave field was found to be rather disordered. The spatial correlation of the collective ciliary motion pattern was found to typically decay within a single wavelength and a broad distribution of propagation directions of metachronal wavelets was found. Therefore, the metachronal wavelength $\lambda$ characterizes the average metachronal wavelength from a rather broad distribution of metachronal wavelets, and was determined by the inverse of the modus of the distribution of the wave vector magnitudes derived from the average spatial power spectrum (Ryser et al. 2007). The distributions of the determined wavelengths are represented in terms of a boxplot for each species in Fig. 7. In mammals, the typical wavelength was found to measure about 50 $\upmu$m, with slightly longer wavelengths in cows and sheeps than in pigs and rabbits. In turkeys and ostriches, however, the wavelength typically measures between 50 and 150 $\upmu$m (see Table 4), with significantly longer wavelengths in turkeys.

**Table 4:** Overview of the determined values for the wavelength $\lambda$ in [$\upmu$m], as inferred from the spatial Fourier analysis

	Cow	Pig	Sheep	Rabbit	Turkey	Ostrich
$\varvec{\lambda }$ [$\varvec{\mu }$m]						
Median	50	46	58	43	121	71
95% CI	[47, 57]	[41, 48]	[54, 65]	[41, 48]	[86, 142]	[56, 84]

The median wavelength and its 95% CI are listed for each species

### Metachronal wave velocity $\left( \vec {v}_w\right)$

**Fig. 8:**
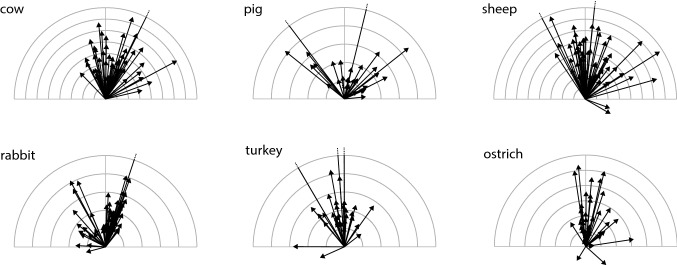
Compilation of mean wave velocities $\vec {v}_w$ from all measured samples of the different species, each vector represents one measurement. The tick interval is 100 $\upmu$m/s

**Fig. 9:**
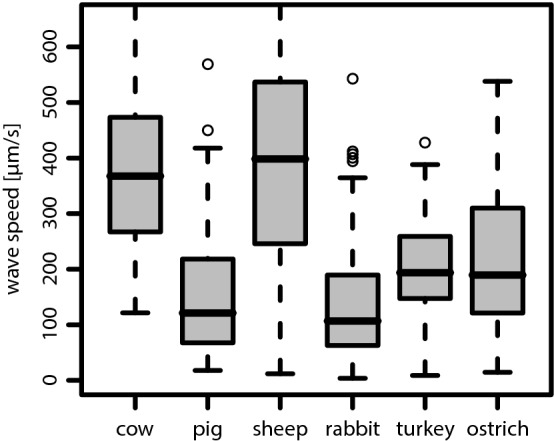
Boxplots of the determined values for the mean wave propagation speed $|\vec {v}_{w}|$

An overview of the mean wave velocities $\vec {v}_w$, as determined by the velocity of the peak correlation in the spatio-temporal correlogram (Ryser et al. 2007), is provided in Table 5 and Fig. 8. The values for the mean wave propagation speed are additionally summarized in the boxplots shown in Fig. 9. Significant differences between species were found. The values for cow and sheep are up to four times higher than those for rabbit and pig. We observed three groups of species with very similar wave velocities. Cow and sheep are distinct from pig and rabbit (Kruskal–Wallis, $p=4.2\cdot 10^{-26}$) and the birds lie in between (Kruskal–Wallis, $p=8.7\cdot 10^{-11}$ and $p=4.3\cdot 10^{-7}$).

**Table 5:** Overview of the determined values for the wave propagation speed (in $\upmu$m/s) and the wave propagation direction (in [$^{\circ }$]) of metachronal waves, as inferred from the spatio-temporal autocorrelation

	Cow	Pig	Sheep	Rabbit	Turkey	Ostrich
$$|\vec {v}_w|$$						
Median	368	121	398	107	194	189
95% CI	[313, 400]	[81, 135]	[325, 446]	[88, 124]	[165, 234]	[149, 255]
Angle						
Median	8.6	26.8	8.4	7.3	$-$ 10.3	$-$ 5.5
CI	[$-$ 1.4, 19.1]	[10.8, 40.3]	[$-$ 1.3, 22.0]	[1.8, 14.6]	[$-$ 16.2, $-$ 0.9]	[$-$ 15.7, 2.1]

The median and its 95% CI of both observables are listed for each species

### Wave propagation direction vs. mucociliary transport direction

As the wave propagation direction and the mucociliary transport direction were simultaneously measured, our data allow to also determine the wave propagation direction in relation to the mucociliary transport direction. Therefore, we determined the angle $\measuredangle \left( \vec {v_t},\vec {v_w}\right)$, which denotes the angle enclosed by the wave propagation direction and the direction of mucociliary transport, for each recording. Table 6 lists the species-specific median values and their corresponding 95% confidence intervals for $\measuredangle \left( \vec {v_t},\vec {v_w}\right)$. It is noticeable that the wave propagates almost along the transport direction and is only slightly deflected to the left (by $-$ 4$^{\circ }$ to $-$ 13$^{\circ }$) in each species.

**Fig. 10:**
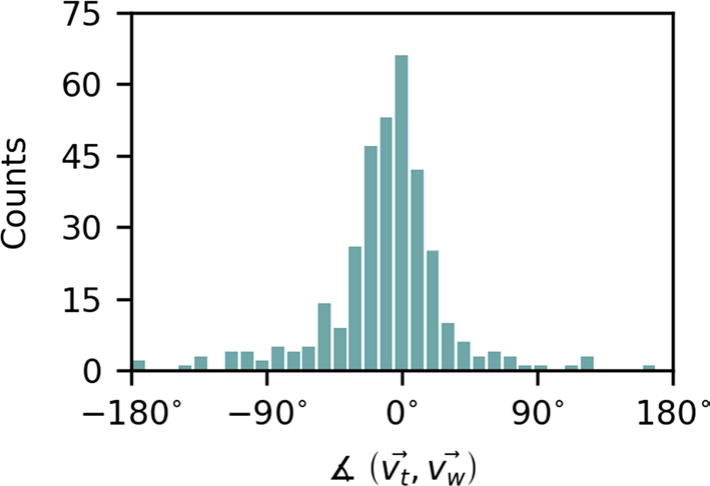
The histogram shows the distribution of the intermediate angle $\measuredangle \left( \vec {v_t},\vec {v_w}\right)$ representing the angle enclosed by the wave propagation direction and the direction of transport over all samples

Figure 10 additionally shows the distribution of the intermediate angle $\measuredangle \left( \vec {v_t},\vec {v_w}\right)$ over all samples. Overall, the histogram shows that the metachronal wave propagation direction is almost aligned with the mucociliary transport direction with a slight tendency to negative values.

**Table 6:** Overview of the determined directions of the wave propagation relative to the direction of transport

	Cow	Pig	Sheep	Rabbit	Turkey	Ostrich
$\varvec{\measuredangle \left( \vec {v_t},\vec {v_w}\right) }$ [$\varvec{^{\circ }}$]						
Median	$-$ 5	$-$ 7	$-$ 4	$-$ 5	$-$ 8	$-$ 13
95% CI	[$-$ 15, 2]	[$-$ 14, 5]	[$-$ 9, 1]	[$-$ 17, $-$ 1]	[$-$ 13, $-$ 2]	[$-$ 21, $-$ 1]

The median wavelength and its 95% CI are listed for each species

### Summary of results and interspecies ranking

**Fig. 11:**
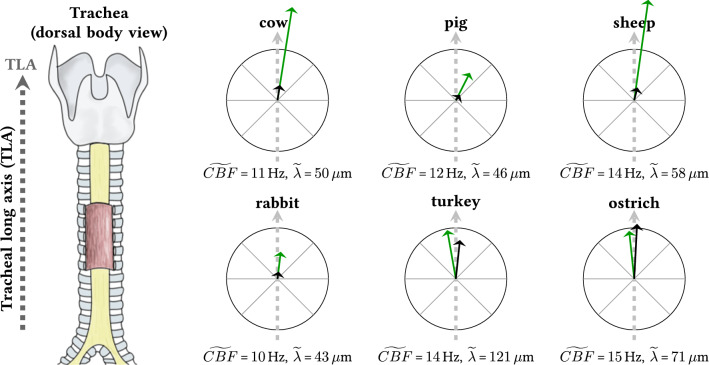
The schematic drawing summarizes the herein presented results. The illustration far left is meant to show that all examined tracheal explants were derived from the center of the anterior tracheal wall. The six black and green arrows shown within the crosshairs on the right represent the mucociliary transport velocity $\vec {v_t}$ (black) and the wave propagation velocity $\vec {v_w}$ (green). For each crosshair, the radius indicates 200 $\upmu$m/s. Therefore, the magnitudes and the directions can directly be compared between species. Furthermore, the numerical values for the median CBF and the median wavelength is provided for each species

The schematic drawing displayed in Fig. 11 concisely summarizes the herein presented results.

Our results clearly reveal that in all six species the mucociliary transport direction strongly correlates with the mean wave propagation direction (compare the black and green arrows in Fig. 11).

The analysis of the determined values for the mucociliary transport speed revealed that the mucociliary transport mechanism in birds is about 3–7 times faster than in mammals (compare the lengths of the black arrows between mammalian and avian species in Fig. 11). Moreover, a rather subtle, but significant, difference in relation to the mucociliary transport direction between birds and mammals was found. While the median mucociliary transport direction was found to significantly deviate from the TLA in all mammalian species, the median transport direction did not significantly deviate from the TLA in birds. Concerning the tubular geometry of the trachea, this means that the mammalian mucociliary transport runs along a left-handed helical trajectory towards the larynx, whereas in birds, it roughly runs straight, along the tracheal long axis.

Surprisingly, although the mucociliary transport speed was found to be clearly higher in birds than in mammals, the analysis of the mean wave propagation speed revealed similar values for birds and mammals. Accordingly, the metachronal waves were found to propagate at about 4–8 times the speed of the mucociliary transport in mammalian species, whereas in avian species, the metachronal waves propagate at about the speed of the achieved mucociliary transport.

Finally, in comparison to mammals, longer metachronal wavelengths were found in birds.

Table 7 summarizes all statistically significant interspecies differences in those observables, which allow for a ranking.

**Table 7:** Summary of all statistically significant interspecies differences in those observables allowing for a ranking

Rank	CBF	Wavelength ($\varvec{\lambda }$)	Wave speed $( |\vec{v}_w|)$	Transport speed $( |\vec {v}_T|)$
1	Ostrich, turkey, sheep	Turkey, ostrich	Sheep, cow	Ostrich
2	Pig, cow	Ostrich, sheep, cow	Turkey, ostrich	Turkey
3	Cow, rabbit	Pig, rabbit	Ostrich, pig, rabbit	Cow, sheep
4				Pig, rabbit

The ranking is based on Wilcoxon–Mann–Whitney tests against a Bonferroni-adjusted level of significance of 0.5%. Species with a different rank have shown significantly different values for the respective observable

## Discussion

High-speed video reflection contrast microscopy was used to simultaneously image the cilia-generated dynamic modulation and transportation of the mucous layer of the ventral tracheal wall in six different species. Our original aim was to establish a generally valid empirical cross-species model for the interrelationship between the characteristics of the dynamic mucociliary wave field and the associated mucociliary transport. Although, such a universal cross-species model could not be established, exciting interspecies differences concerning the mucociliary clearance mechanism could be revealed. As the mucociliary transport velocity represents the most important observable, we shall take the herein presented results for this observable as the starting point for our discussion.

As summarized in the preceding section, the tracheal mucociliary transport in all examined mammalian species was found to run along a left-handed helical trajectory towards the larynx, whereas in avian species, the direction of transport does not significantly differ from the tracheal long axis. The difference between mammalian and avian species was even more pronounced, when concerning the mucociliary transport speed: in average, birds were found to exhibit 3–7 times higher mucociliary transport speeds than mammals.

The finding of a left-handed mucociliary transport in the mammalian trachea agrees well with previous observations of the mucociliary transport direction in the trachea of cats (Barclay et al. 1937), dogs (Sackner et al. 1973), humans (Santa Cruz et al. 1974; Nakamura et al. 2004), pigs and sheeps (Donnelley et al. 2017). In the latter study, performed by Donnelley et al. (2017), synchrotron X-ray imaging was used to examine the tracheal mucociliary clearance in pigs and sheeps. The experiments were performed at 37 $^{\circ }$C and the tracer particles were an order of magnitude larger than the ones used here. Donnelley et al. reported the mucociliary transport mechanism in the pig trachea to be considerably more circumferential than in the sheep trachea. In view of the fundamentally different observation techniques, the accordance to our results concerning the mucociliary transport velocity in the trachea of pigs and sheeps is remarkable (compare to Table 2).

Our numerical values obtained for the average tracheal mucociliary transport speed in mammals, roughly ranging from 20–80 $\upmu$m/s, are in good accordance with previously published mean values: 27 $\upmu$m/s in sheeps (Donnelley et al. 2017), 22 $\upmu$m/s in pigs (Donnelley et al. 2017) and 53 $\upmu$m/s in rabbits (Felicetti et al. 1981).

Our numerical results for the average CBF, which roughly ranges from 10 to 15 Hz, agree well with previously published data. In a comparative study on CBF, Joki and Saano (1994) also observed small interspecies variations. Since their measurements were performed at 37 $^{\circ }$C, however, they reported slightly higher CBF values for bovine, porcine and lapine tracheas. It is known that the temperature is one of the key parameters determining the frequency of the ciliary oscillations (Kurosawa et al. 1995; Mercke et al. 1974).

Here we found typical mean metachronal wavelengths of around 50 $\upmu$m in mammals. A tendency towards considerably longer wavelengths of around 100 $\upmu$m was found on the trachea explants of the two examined avian species. Previous studies performed on other mucus-transporting ciliated tissues have reported markedly shorter wavelengths. In Yi et al. (2000), thin healthy human samples of mucosa were collected from the sphenoid sinus. After adding culture medium solution, the samples were placed in a temperature-controlled chamber maintained at 37 $^{\circ }$C and imaged by high-speed video transmission microscopy. The video analysis, which is based on correlation maps, yielded metachronal wavelengths of approximately 6 $\upmu$m. In cultured frog esophagus epithelium, the metachronal wavelength was found to range from 5 to 9 $\upmu$m (Gheber and Priel 1994). Finally, Machemer (1972) found the metachronal wavelength on the ciliated surface of forward swimming paramecium to lie between 13 and 16 $\upmu$m; at a temperature of 20 $^{\circ }$C and a viscosity of 40 cP. Although, a comparison to the frog esophagus epithelium and to the surface of paramecium is often made, it is hardly justified, as these model tissues are structurally as well as functionally different. The much shorter wavelengths observed by Yi et al. (2000) on nasal mucosa samples, may result from several factors. First, bathing the mucosa in culture medium leads to a drastic decrease of the mucus viscoelasticity. Second, it is likely that the cilia are more aligned in the tracheal mucosa than in epithelium samples derived from the nasal sinuses. Finally, the observations presented in Yi et al. (2000) were made at 37 $^{\circ }$C. An increase in temperature has recently been shown to decrease the mucus viscosity as well as elasticity on human bronchial epithelium cultures (Jory et al. 2019; Humphries 2013).

In this study, the most fundamental finding—when considering the tracheal mucociliary clearance mechanism in its entirety—is the strong correlation between the mean wave propagation direction and the mucociliary transport direction found in all six species. Therefore, our results do not confirm the generally accepted picture of antiplectic or antilaeoplectic metachronism, which would mean wave propagation antiparallel to the effective stroke (i.e. antiparallel to the transport direction). We found the mean wave speed to roughly range from 100 to 400 $\upmu$m/s. Interestingly, the metachronal waves were found to propagate at about 4–8 times the speed of mucociliary transport in mammalian species, whereas in birds, the metachronal waves were found to propagate at about the speed of the achieved mucociliary transport.

Overall, the mucociliary transport was found to be 3–7 times faster in birds than in mammals and one naturally asks where this difference comes from. The interspecies differences found in terms of CBF are rather small and cannot explain the observed bird/mammal-difference in transport speed. The tendency towards increased wavelengths, as well as the decreased ratio between the speed of the metachronal wave and the mucociliary transport speed, indicate changes in the spatial structure of the wave field in birds as well as that the metachronal wave field is differently related to the achieved mucociliary transport in the avian than in the mammalian trachea.

The majority of biological rates are known to exhibit temperature dependence (Humphries 2013) and therefore, the body temperature represents an important observable characterizing the general physiology. However, the species-specific body temperatures (listed in Table 8) hardly seem to have any connection with the observed bird/mammal-differences. Our results thus indicate, that the mucociliary clearance mechanism functions differently in birds than in mammals. Some gross anatomical differences are obvious and may be related: birds ventilate their lungs using air sacs and their tracheas are on average 2.7 times longer and about 1.3 times wider than those of comparably sized mammals (Hinds and Calder 1971). Further, avian tracheas have full cartilage rings, instead of the C-shaped ones, which are connected by smooth muscle found in mammalian tracheas. Consequently, avian airways cannot contract and the effectiveness of cough clearing is drastically reduced. These facts emphasize the need for a more effective clearing system in birds and may be responsible for a certain evolutionary pressure. Brown et al. (1997) even suggest, that a novel mechanism assisting clearance is needed, when the tracheal length approaches 10 times the length of comparably sized mammals (which is nearly true for ostriches, which were found to exhibit the highest clearance speed)—giraffes would therefore be of interest.

**Table 8:** Body temperatures of the examined species

	Cow	Pig	Sheep	Rabbit	Turkey	Ostrich
Body temp. [${^\circ }$C]	38.3–39.4	38.5	39.0	39.6	41.2	37.2–38.1 & 38.7
Source	(Atkenson and Bickert 1997)	(Noffsinger and Andrews 1959)	(Johnson 1987; Laburn et al. 1992)	(Simpson and Galbraith 1905)	(King and Farner 1961)	(Schrader et al. 2009; Bligh and Hartley 1965)

As a general empirical cross-species model mathematically capturing the relationship between the spatio-temporal properties of the metachronal wave field and its associated mucociliary transport could not be established, we conclude that, in order to establish such a general model for mucociliary transport, further observables determining mucociliary transport need to be taken into account. The metachronal wave properties are commonly considered to self-organize out of local hydrodynamic interactions as well as to co-emerge with mucociliary transport (Brumley et al. 2014; Schneiter et al. 2019). As the metachronal wave properties (e.g. Gheber et al. 1998) as well as the mucociliary transport speed (e.g. Sedaghat et al. 2016) are likely governed by the rheological mucus properties, we suspect that those are different for each species. This would explain, why our attempt to establish a generally valid model for the interrelationship of the metachronal wave properties and the mucociliary transport was not successful. Besides the rheological conditions (Norton et al. 2011; Sedaghat et al. 2016), the geometrical conditions, such as the ciliary spacing (Elgeti and Gompper 2013; Lee et al. 2011), the ciliary orientation (Schneiter et al. 2021), the proportion and the distribution of ciliated cells (Plopper et al. 1983; Ramirez-San Juan et al. 2020), but also the depth of the mucus and the periciliary layer (Lee et al. 2011), are known to also determine mucociliary transport, which, in turn, is contemporary seen to interact and to co-emerge with those rheological and geometrical conditions during the morphogenesis of the system under study.

## Conclusions

The mucociliary transport velocity, the ciliary beating frequency, the metachronal wavelength and the metachronal wave velocity were simultaneously determined on tracheal epithelium derived from six different species. In each species, the mean wave propagation direction strongly correlates with the direction of mucociliary transport, meaning that the tracheal cilia in cows, pigs, sheeps, rabbits, turkeys and ostriches predominantly exhibit a symplectic metachronism. The tracheal mucociliary transport was found to be considerably faster in avian than in mammalian tracheas. Furthermore, in mammals, the mucociliary transport runs along a left-handed helical trajectory in pharyngeal direction, whereas in birds, it roughly runs straight along the tracheal long axis. Compared to mammals, birds show a considerably lower ratio of the metachronal wave speed and the mucociliary transport speed as well as a tendency towards longer metachronal wavelengths. Therefore, the mucociliary clearance mechanism likely operates slightly differently in birds than in mammals, which we suspect is mainly due to differences in the composition and/or the structure of the airway surface liquid.

In addition to the simultaneous measurement of the metachronal wave field and the mucociliary transport velocity, future attempts for the establishment of a generally valid cross-species model for mucociliary clearance, need to at least take the in situ thickness and viscoelasticity of the mucus into account.

## Supplementary Information

Below is the link to the electronic supplementary material.

Supplementary file1 (MP4 1271 KB)

Supplementary file2 (MP4 1692 KB)

Supplementary file3 (MP4 2432 KB)

Supplementary file4 (MP4 1604 KB)

## Data Availability

The datasets used and analyzed during the current study are available from the corresponding author upon reasonable request.

## Footnotes

1 This was also the motivation for including the data measured for ostrich, even if the number of individuals is much lower than for the other species.
